# The overlap between complex posttraumatic stress disorder and borderline personality disorder

**DOI:** 10.1192/j.eurpsy.2021.1997

**Published:** 2021-08-13

**Authors:** C. Álvarez García, L. Nocete Navarro, M.D.C. Molina Lietor, I. Cuevas Iñiguez, A. Sanz Giancola

**Affiliations:** 1 Psychiatry, Hospital Universitario Príncipe de Asturias, Alcalá de Henares, Spain; 2 Psiquiatría, Hospital Universitario Príncipe de Asturias, Alcalá de Henares, Spain

**Keywords:** cPTSD, BPD, overlap

## Abstract

**Introduction:**

Research has shown the relationship between borderline personality disorder (BPD) and complex posttraumatic stress disorder (cPTSD), pointing out the overlapping nature and expression of both conditions. In order to understand their differences and similarities, we present a case of a 22-years-old patient with a history of repeated sexual trauma throughout all her adolescence, whose diagnose was changed from BPD to cPTSD after she was admitted in an acute inpatient mental health unit.

**Objectives:**

To gather the similarities between borderline personality disorder and complex posttraumatic stress disorder.

**Methods:**

A narrative review of the literature through the presentation of a case. Articles were chosen based on its clinical relevance.

**Results:**

cPTSD merges the clinical features and symptoms of PTSD with affect dysregulation, negative self-perception, unstable relationships and somatization, also present in BPD. Furthermore, BPD is known to frequently have a traumatic etiology.

**Conclusions:**

It is not always simple to draw a clear line between cPTSD and BPD conditions. However, each diagnosis may have a different impact on patient understanding and treatment.

**Disclosure:**

No significant relationships.

